# Geographic Distribution of HCV-GT3 Subtypes and Naturally Occurring Resistance Associated Substitutions

**DOI:** 10.3390/v11020148

**Published:** 2019-02-11

**Authors:** Sabrina Bagaglio, Emanuela Messina, Hamid Hasson, Andrea Galli, Caterina Uberti-Foppa, Giulia Morsica

**Affiliations:** 1 Division of Infectious Diseases, Ospedale San Raffaele, 20132 Milan, Italy; messina.emanuela@hsr.it (E.M.); hasson.hamid@hsr.it (H.H.); galli.andrea@hsr.it (A.G.); morsica.giulia@hsr.it (G.M.); 2 Vita-Salute University, 20132 Milan, Italy; uberti.caterina@hsr.it

**Keywords:** NS5A replication complex inhibitors, resistance-associated substitutions (RASs), HCV genotype 3 subtypes, geographic distribution, natural polymorphisms

## Abstract

**Background:** Little is known about the frequency or geographic distributions of naturally occurring resistance-associated substitutions (RASs) in the nonstructural protein 5A (NS5A) domain of hepatitis-C virus (HCV) genotype-3 (GT-3) different subtypes. We investigated naturally occurring GT-3 RASs that confer resistance to NS5A inhibitors. **Methods:** From a publicly accessible database, we retrieved 58 complete GT-3 genomes and an additional 731 worldwide NS5A sequences from patients infected with GT-3 that were naive to direct-acting antiviral treatment. **Results:** We performed a phylogenetic analysis of NS5A domains in complete HCV genomes to determine more precisely HCV-GT-3 subtypes, based on commonly used target regions (e.g., 5′untranslated region and NS5B partial domain). Among 789 NS5A sequences, GT-3nonA subtypes were more prevalent in Asia than in other geographic regions (*p* < 0.0001). The A30K RAS was detected more frequently in HCV GT-3nonA (84.6%) than in GT-3A subtypes (0.8%), and the amino acid change was polymorphic in isolates from Asia. **Conclusions:** These results provided information on the accuracy of HCV-3 subtyping with a phylogenetic analysis of the NS5A domain with data from the Los Alamos HCV genome database. This information and the worldwide geographic distribution of RASs according to HCV GT-3 subtypes are crucial steps in meeting the challenges of treating HCV GT-3.

## 1. Introduction

Hepatitis C virus (HCV) is classified into seven genotypes (GT-1 to GT-7) and 67 subtypes. HCV genotypes display about 30% divergence and HCV subtypes display about 15% divergence [1]. Genotype-1 (GT-1) accounts for 46% of infections, followed by GT-3 (22%) [2]. HCV GT-1 and GT-3 are distributed worldwide, and GT-3 is endemic in Asia (39% of all infections). Among the GT-3 subtypes, GT-3A is most prevalent in North America (98.7%), Europe (98.9%), and Oceania (98.7%). In India, about 56% of patients are infected with GT-3A; however, other subtypes, including 3B (20.3%), 3G (6.8%), and 3I (3.6%), are also present in that population [3].

HCV genotypes and subtypes are major determinants for the selection and duration of direct-acting antiviral (DAA) treatment regimens. In this context, current HCV genotyping assays are limited in the precise assignment of genotype and subtype, which can result in suboptimal treatment [4]. Another important and debated potential virological factor that may negatively influence the response to DAAs is the presence of baseline RASs. Nonstructural protein 5A (NS5A) RASs with amino acid substitutions M28T, A30K, L31M/V, and Y93H, are often detected at baseline in patients with GT-3 that are DAA-naive. Consequently, baseline resistance testing is currently recommended in the USA [5] before initiating HCV treatment with NS5A inhibitors in all patients with compensated cirrhosis that either have or have not had prior experience with GT-3A treatments.

In Europe [6], NS5A baseline resistance testing is not recommended before treating GT-3A with NS5A inhibitors. When resistance testing is performed and specific RASs are detected, it is mandatory to extend the duration of DAA treatment and/or concomitantly administer ribavirin. In this context, because NS5A-resistance testing for GT-3 can be technically challenging, European guidelines indicate that a reliable result is not guaranteed in all cases.

Currently, little information is available on the geographic distribution of RASs, according to GT-3 subtype. For example, it is well known that RAS Y93H is naturally present in GT-3A, but no data are available on the frequency of this RAS in other GT-3 subtypes. One report from China [7] showed that A30K was present in 14/48 GT-3 natural sequences. Hernandez et al. [8] derived 96 sequences from patients infected with GT-3 that were treatment-naive. Those patients were from North America, Europe and Australia. The predominant variant was NS5A A30 (85/96 sequences, 89%), but the A30K variant was detected in 6/96 sequences (6.3%) and A30L variants existed at lower frequencies (two sequences). They also found the Y93H at a frequency of 8.3% (8/96 sequences). However, those two studies did not detail the frequencies of RASs, according to the GT-3 subtype.

The response rates to currently available DAAs are lower in patients with GT-3, particularly those with cirrhosis or have prior treatment experience, compared to patients infected with other genotypes [9,10]. Moreover, limited data are available on the presence and distribution of RASs in the NS5A domain of HCV GT-3 subtypes. Therefore, we aimed to examine the frequency of naturally occurring RASs and to characterize their natural polymorphisms, according to their geographic distributions and GT-3 subtypes.

## 2. Materials and Methods

### 2.1. Design of the Study and Methodological Approach

**Figure 1:**
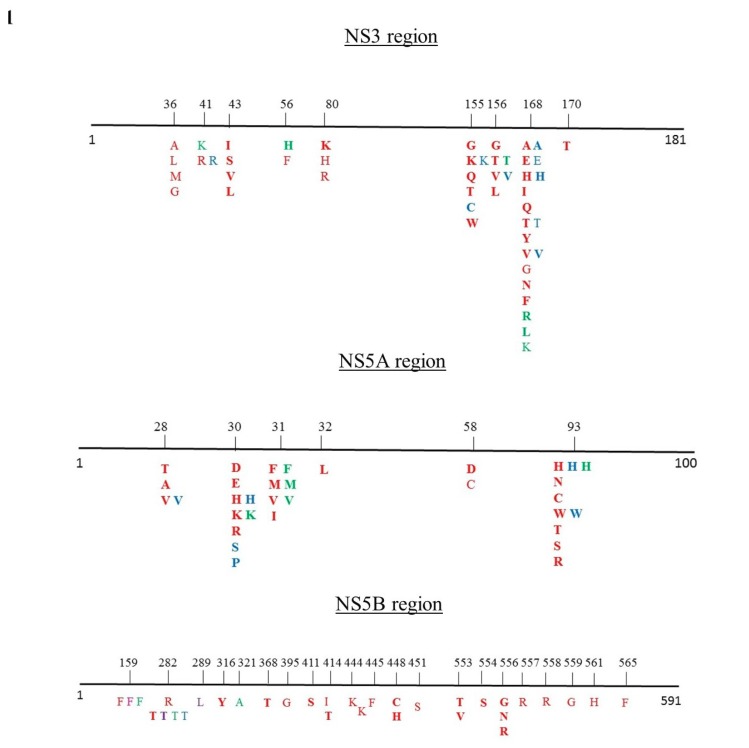
NS3, NS5A and NS5B resistance-associated mutation sites previously identified [11,12,13] and corresponding amino acid substitutions predicted with the Geno2Pheno algorithm. Substitutions are color-coded, based on genotype; red: GT-1; violet: GT-2; green: GT-3; blue: GT-4. Substitutions that confer resistance, according to Geno2Pheno classifications, are indicated in bold. The other substitutions reduced the susceptibility to NS5B inhibitors.

First, we examined the presence of RASs within the nonstructural protein 3 (NS3) protease, NS5A and nonstructural protein 5B (NS5B) polymerase domains in 58 complete GT-3 genomes that were deposited in the Los Alamos HCV database before 2013, with available geographic origin data. To ensure the quality of the data, public sequences were excluded from the analysis when they contained stop codons. Multiple sequences from the same patient and recombinant or clonal sequences were excluded from the analysis. Mutation analysis was performed at the resistance-associated positions in NS3, NS5A, and NS5B [11,12,13]. RAS profiles were identified by reviewing the literature [11,12,13] and applying the Geno2Pheno HCV algorithm. A schematic representation of the RAS positions is shown in Figure 1.

**Figure 2:**
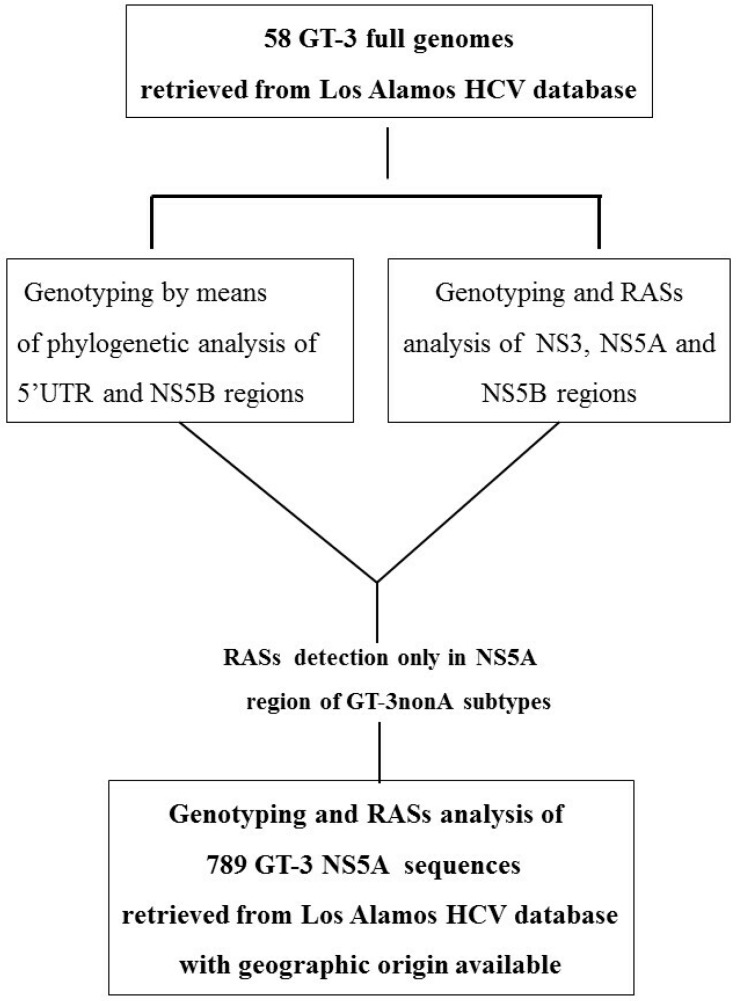
Schematic representation of the study design.

Next, in the same isolates, we compared the concordance of GT-3 subtypes identified in a phylogenetic analysis of a 5′untraslated (UTR) region, a partial NS5B region (which is commonly used in routine laboratory testing for genotyping), and the DAA target regions of NS3, NS5A, and NS5B. Among these 58 isolates, no RASs were found in the NS3 or NS5B domains, but 11/58 (19%) isolates harbored the A30K RAS and clustered with GT-3nonA. Therefore, we decided to extend the analysis to NS5A sequences deposited before 2010 into the Los Alamos HCV database [14]. We selected sequences derived from patients infected with HCV GT-3 that were naive to NS5A inhibitor treatments and had geographic origin data available. The study design is summarized in Figure 2. The accession numbers of the sequences included in the analysis are summarized in Tables S1 and S2. Amino acid changes were considered polymorphic or non-polymorphic, when present in at least 10% or <10% of sequences, respectively.

### 2.2. Phylogenetic Analysis of 5′UTR, NS3, NS5A, and NS5B Domains

We applied Kalign to obtain a multiple alignment of nucleotide sequences (5′UTR, NS3, NS5A, and NS5B) for 58 isolates with full genomes available from the Los Alamos HCV database and a multiple alignment of the larger set of NS5A sequences. Kalign employs the Wu–Manber string-matching algorithm to improve both the accuracy and speed of multiple sequence alignment. Compared to the best of other methods, Kalign had similar accuracy for small alignments, but significantly better accuracy for large and distantly related sets of sequences. Kalign is a fast robust alignment method that was particularly well suited for aligning large numbers of sequences. The output file from the Kalign analysis is called a ClustalW file.

We calculated nucleotide distances by generating a distance matrix with the maximum-likelihood model available in the DNADIST program (PHYLIP 3.5c package). A phylogenetic tree was then constructed with the NEIGHBOR (PHYLIP) program, with random addition. The phylogenetic tree was drawn with TreeViewPPC, version 1.5.3. We performed a bootstrap analysis with SEQBOOT (100 rounds of resampling) to place approximate confidence limits on individual nodes. Bootstrap values ≥75 are indicated.

### 2.3. Selective Pressure Analysis on NS5A Domain 1

To analyze selective pressure on each codon of NS5A domain 1 region (aa 1–100: target region of NS5a inhibitors) we applied data monkey tools to our data set. Specifically we run Mixed Effects Model of Evolution (MEME) algorithm. MEME employs a mixed-effects maximum likelihood approach to test the hypothesis that individual sites have been subject to episodic positive or diversifying selection. In other words, MEME aims to detect sites evolving under positive selection. This model estimates a site-wise synonymous (α) and a two-category mixture of nonsynonymous (β-, with proportion p-, and β+ with proportion 1-p-) rates, and uses a likelihood ratio test to determine if β + > α at a site [15]. A *p* value < 0.05 was considered as statistically significant.

### 2.4. Statistical Analysis

Data were analyzed with a chi-squared or Fisher’s exact test, when appropriate. A *p*-value < 0.05 was considered statistically significant.

## 3. Results

### 3.1. Concordance of GT-3 Subtypes Resulting from Phylogenetic Analysis of Different Regions (5′UTR, NS3, NS5A, and NS5B) in 58 Isolates Obtained from Los Alamos HCV Database

**Figure 3:**
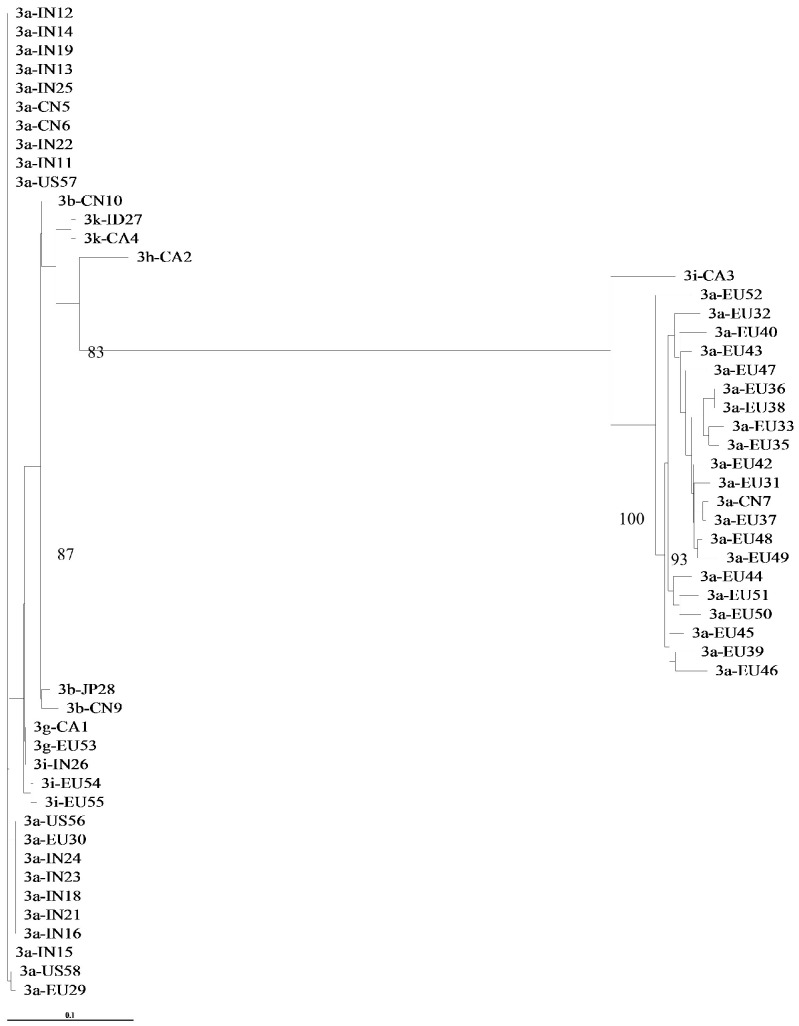
Phylogenetic tree of the 5′UTR region of 53 complete genomes retrieved from Los Alamos database. The maximum-likelihood phylogenetic rooted tree shows the relationships between different GT-3 subtypes of 53 isolates that had 5′UTR sequences available for analysis. Evolutionary distances are indicated next to the branches. Two isolates (CA2, CA3) clustered with GT-3A, but were identified as GT-3I and GT-3H subtypes, respectively, in a NS3, NS5A and NS5B phylogenetic analysis.

**Figure 4:**
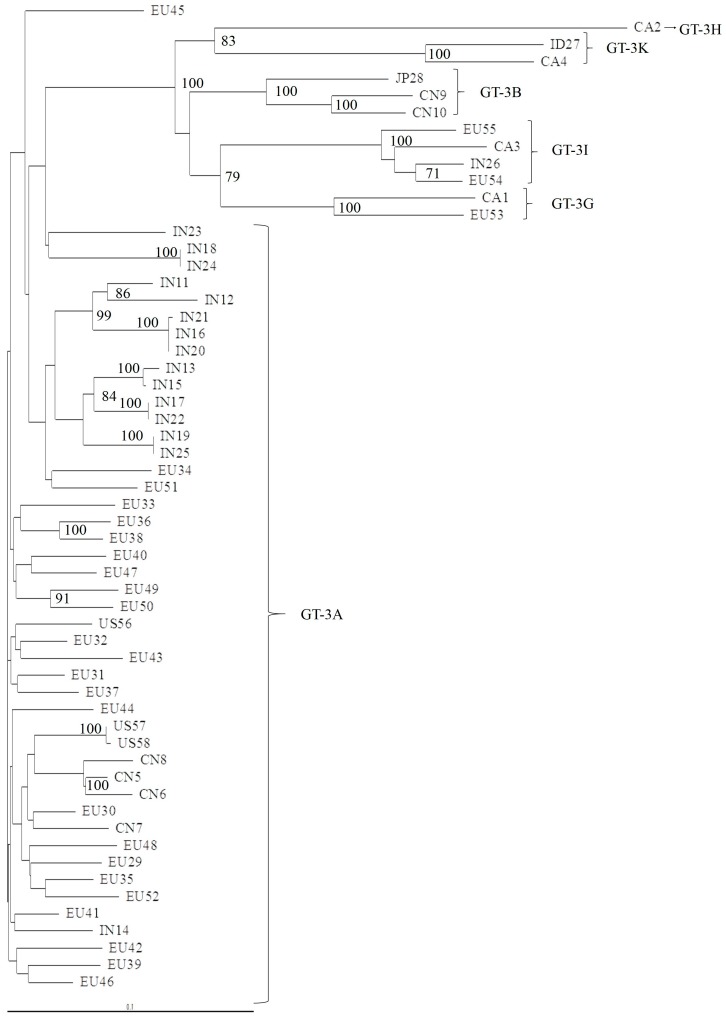
Phylogenetic tree of the NS3 region of 58 complete genomes retrieved from the Los Alamos database. The maximum-likelihood phylogenetic rooted tree of the NS3 protease region shows the relationships between different GT-3 subtypes of 58 isolates that had full genomes available. The geographic origin of each isolate is indicated with a code, followed by a progressive number. The geographic codes are: BR = Brazil, EU = Europe, US = USA, AU = Australia, CA = Canada, PK = Pakistan, IN = India, TH = Thailand, CN = China, ID = Indonesia, and JP = Japan. Evolutionary distances are indicated next to the branches.

**Figure 5:**
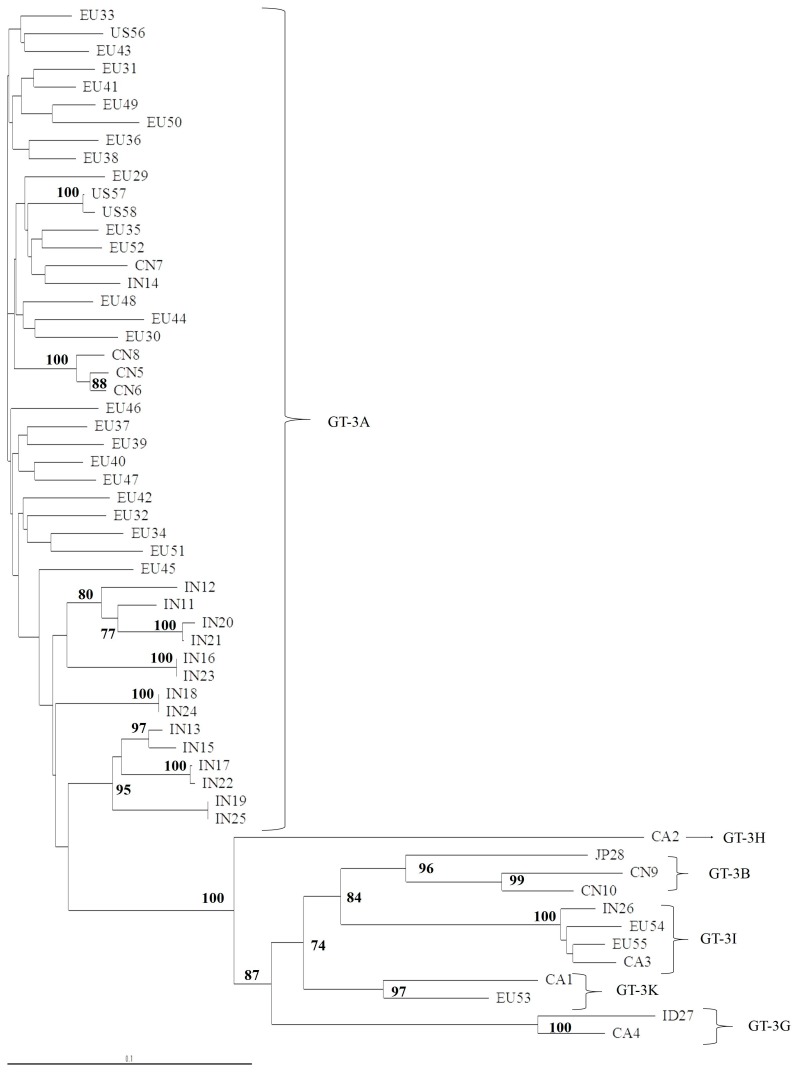
Phylogenetic tree of NS5A domain 1 of 58 complete genomes retrieved from the Los Alamos database. The maximum-likelihood phylogenetic rooted tree of NS5A domain 1 shows the relationships between different GT-3 subtypes of 58 isolates that had full genomes available. The geographic origin of each isolate is indicated with a code, followed by a progressive number. The geographic codes are: BR = Brazil, EU = Europe, US = USA, AU = Australia, CA = Canada, PK = Pakistan, IN = India, TH = Thailand, CN = China, ID = Indonesia, and JP = Japan. Evolutionary distances are indicated next to the branches.

**Figure 6:**
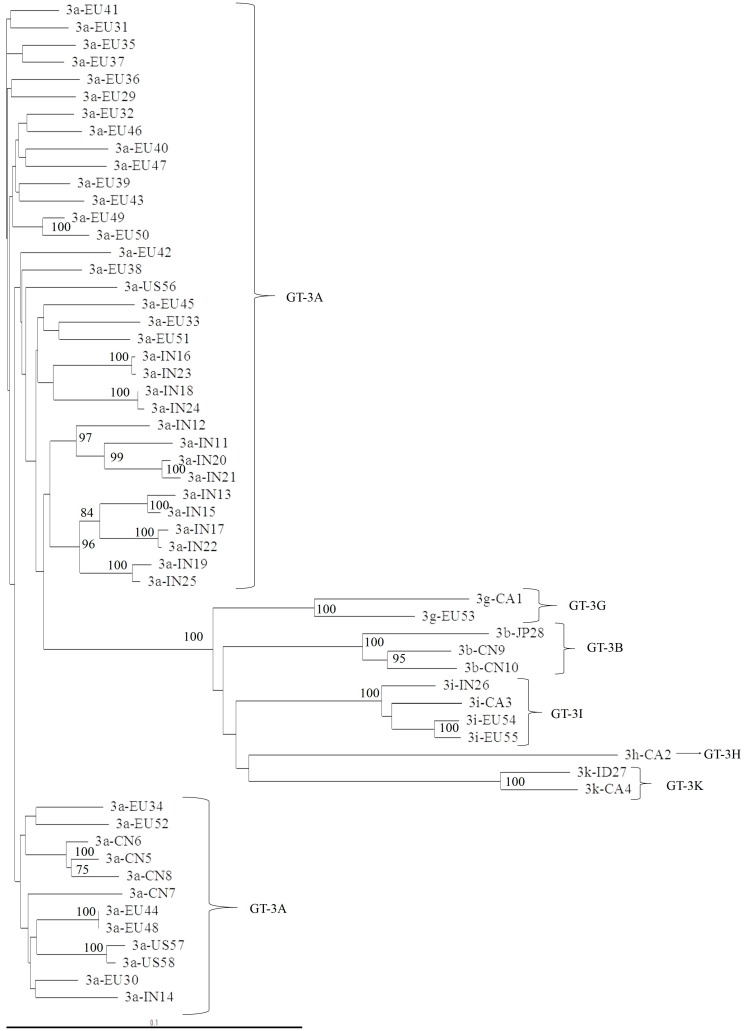
Phylogenetic tree of the NS5B region of 58 complete genomes retrieved from the Los Alamos database. The maximum-likelihood phylogenetic rooted tree of the NS5B region shows the relationships between different GT-3 subtypes of 58 isolates that had full genomes available. The geographic origin of each isolate is indicated by a code, followed by a progressive number. The geographic codes are: BR = Brazil, EU = Europe, US = USA, AU = Australia, CA = Canada, PK = Pakistan, IN = India, TH = Thailand, CN = China, ID = Indonesia, and JP = Japan. Evolutionary distances are indicated next to the branches.

We found that in GT-3, subtype determination was concordant for NS3, NS5A, and NS5B, but the subtype characterization was not precise when we used a phylogenetic analysis of the 5′UTR region (Figure 3). For example, two isolates clustered with GT-3A in a phylogenetic analysis of 53 available 5′UTR sequences, but these isolates were identified as GT-3I or GT-3H, in the NS3 (Figure 4), NS5A (Figure 5), and NS5B (Figure 6) analyses. Moreover, the latter subtypes were concordant with those recorded in the Los Alamos report and confirmed with the Geno2Pheno algorithm.

### 3.2. NS5A Analysis and Geographic Distribution, According to GT-3 Subtypes

In the Los Alamos HCV database, we found 789 sequences that belonged to GT-3: 763 (96.7%) isolates clustered with GT-3A and the remaining 26 (3.3%) clustered with GT-3nonA. Details on the geographic distribution of GT-3 subtypes are shown in Table 1.

We compared the frequency of GT-3A vs. GT-3nonA subtypes between Asian isolates and isolates from other geographic regions (non-Asian isolates were from North America, Australia, Europe, and Brazil). We found that, of 80 (77.5%) sequences from Asia, 62 belonged to GT-3A and 18 (22.5%) belonged to GT-3nonA. In contrast, among the 709 non-Asian sequences, 701 (98.8%) belonged to GT-3A and eight (1.2%) belonged to GT-3nonA, *p* < 0.0001.

### 3.3. NS5A RASs According to Geographic Distribution and Subtypes 

**Figure 7:**
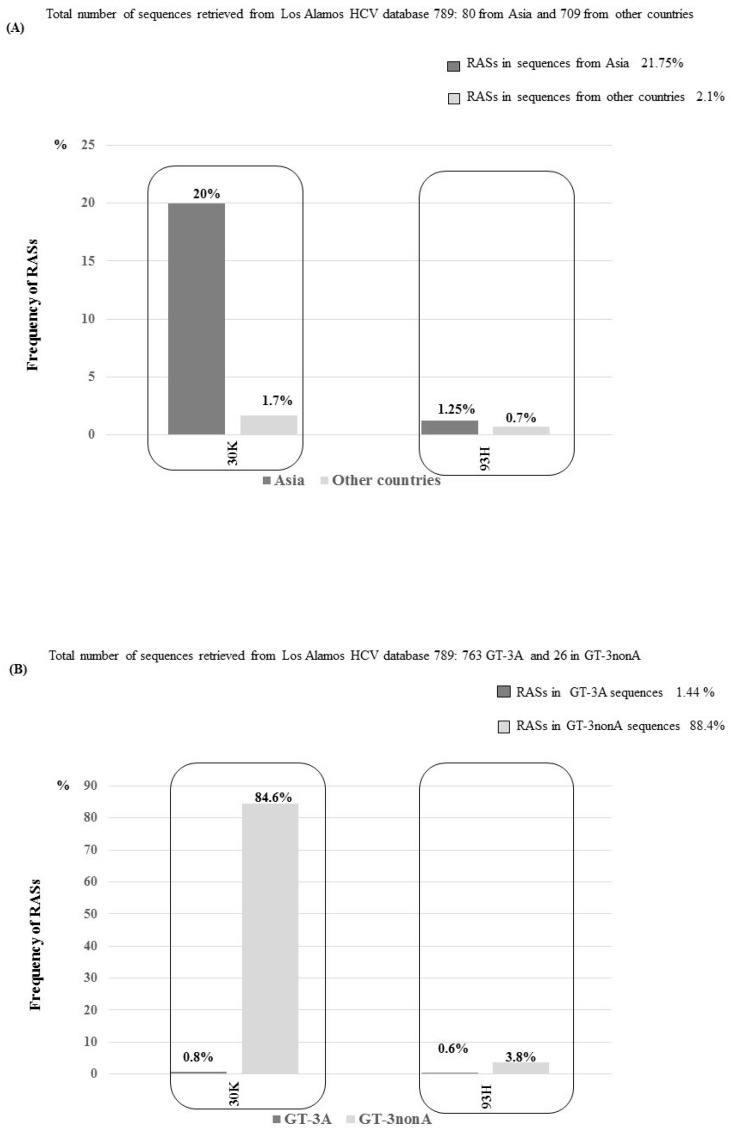
Frequencies and geographic distributions of the A30K and Y93H substitutions in the NS5A region. (**A**) Frequencies of A30K and Y93H according to geographic origin of isolates (Asia vs. other countries). (**B**) Frequencies of A30K and Y93H according to GT-3 subtype (GT-3A vs. GT-3nonA).

An analysis of 789 sequences showed that NS5A RASs were present in 32 isolates (4%). The majority of mutations were found in Asian isolates (17/80 sequences, 21.25%); only 15/709 (2.1%) were found in non-Asian sequences (Figure 7).

Interestingly, the RAS A30K showed a polymorphic profile in isolates from Asia (16/80, 20%), but not in non-Asian isolates (12/709, 1.7%), *p* < 0.0001 (Figure 7). This RAS was also more frequently detected in GT-3nonA than in GT-3A isolates. We detected the A30K substitution in 22/26 (84.6%) GT-3nonA sequences, including 14/16 (87.5%) GT-3B isolates, 2/2 (100%) GT-3G sequences, 2/3 (66%) GT-3K isolates, and 4/5 (80%) GT-3I sequences. Only 6/763 (0.8%) GT-3A isolates harbored this RAS (*p* < 0.0001), which indicated that the majority of GT-3nonA isolates naturally harbored the A30K, rather than the 30A, substitution (Figure 7). Interestingly, all GT-3A isolates that harbored the A30K RAS were from Brazil, and of these six resistant strains, two harbored both the A30K and Y93H RASs. Another amino acid substitution, 30L, was found to be polymorphic in Asia, but not in other countries. This substitution was exclusively detected in GT-3A Asian isolates (12/62, 19.3% sequences had A30L) and not in GT-3A non-Asian isolates, *p* < 0.0001 (Figure 7). Of 12 sequences that harbored A30L, 11 were from Pakistan and one was from India. Also at position 30, in sequences from Asia, we detected an A30R substitution (one GT-3B sequence) and a A30T substitution (three GT-3A sequences). In non-Asian isolates, we observed a A30M (one GT-3A sequence), a A30V (two GT-3A sequences), and a A30R (one GT-3I sequence) substitution.

Finally, the RAS Y93H substitution was detected in one isolate from Asia (Thailand) that belonged to the GT-3nonA group and in five non-Asian sequences that clustered with GT-3A. Of the non-Asian sequences, four were from Brazil and one was from Australia. Two of these five sequences harbored both the A30K and Y93H substitutions. Fold change and replicative capacity of variant harboring A30K, Y93H and A30K+ Y93H RASs are summarized in Table 2. In detail, fold change of A30K and Y93H, considering Daclatasvir [8] or Pibrentasvir [16], were obtained by using hybrid chimeric replicons in which the sequence of NS5A region belonged to GT-3A and considering Velpatasvir, by using GT-3A replicon S52 [17].

### 3.4. Selective Pressure on NS5A Domain 1 According to Subtype and Geographic Origin

The application of MEME algorithm to detect selective pressure on NS5A domain 1 showed that the codon at position 62 was under selective pressure in GT-3A regardless of geographic origin of isolates. In detail: the Serine (S) at position 62 was replaced by a Threonine (T) in 16.8% of GT-3A sequences and the other positively selected substitutions at this position were Alanine (A), Proline (P), Leucine (L), Methionine (M) and Aspartic acid (D). The codon at position 30 showed a selective pressure according to subtype and geographic origin of isolates [(Asian GT-3nonA isolates) (Table 3)]: the Alanine (A) at position 30 was frequently replaced by Lysine (K). In one GT-3B isolate from China, an Arginine (R) was observed at position 30.

## 4. Discussion

In the present study, we initially performed an analysis of the NS3, NS5A, and NS5B domains of HCV GT-3 isolates retrieved from an international database to identify the frequency of naturally occurring RASs. We found that the NS3 and NS5B domains in GT-3 were highly conserved. In contrast, we found that the NS5A domain was less conserved, with regard to RASs, and that these mutants were more frequently detected in GT-3nonA than in GT-3A isolates. Additionally, a phylogenetic analysis of different HCV regions showed that sequences in the 5′ UTR amplicons provided less precise subtype identification compared to other regions. Our analysis provided precise determinations of GT-3 subtype-related RASs, and their prevalence, according to geographic origin.

Our analysis of the geographic distribution of GT-3 subtypes revealed that a number (26.5%) of isolates from Asia clustered with GT-3nonA, and the majority of non-Asian sequences (99.4%) clustered with GT-3A. An analysis of the distribution of RASs, according to HCV subtype and geographic origin, revealed that isolates from Asia displayed a high frequency of RASs and amino acid substitutions in sites of resistance.

In particular, we showed that the A30K RAS and the A30L amino acid substitution were polymorphic in isolates from Asia, but not in non-Asian sequences. Additionally, the A30K RAS occurred more frequently in GT-3nonA subtypes (85%) than in GT-3A subtypes.

The A30L substitution was detected in GT-3A Asian sequences (19%), but not in GT-3A non-Asian isolates. Thus, our data were consistent with findings from Welzel et al. [3], who showed that about 1.3% of non-Asian isolates clustered with GT-3nonA, and 22.8% of Asian sequences clustered with GT-3nonA. That same study showed that 30A was prevalent in GT-3A (91%) isolates, and A30K was more frequently detected (96–100%) in GT-3nonA isolates (except subtype 3H, where the 30A was found in 100% of sequences). The A30L substitution was not described by Welzel et al. [3].

One other previous study also evaluated the worldwide distribution of RASs, including six main genotypes (GT-1 to 6) [18]. They showed that about 80% of GT-3 isolates harbored 30A, 13% harbored A30K, and 1% harbored A30L. However, that study did not provide information on the RAS distribution at the subtype level or their geographic origins.

Hernandez et al. [8] showed that 8.3% of clinical strains with different geographic origins naturally harbored Y93H, but they found a lower frequency (1.3%) of this RAS in isolates in a European HCV database. Consistent with the frequency they found in the European HCV database, in the present study, we found a low rate (0.75%) of Y93H in GT-3 sequences from the Los Alamos database, and its presence was not related to a specific subtype.

The effect of a pre-existing RAS on NS5A inhibitor activity was examined in a clinical trial (ALLY-3) that investigated sofosbuvir/daclatasvir (sof/dacl) HCV treatments [19]. They showed that, among 14 patients infected with HCV GT-3 that had pre-existing 30A polymorphisms, 9/9 (100%) without cirrhosis and 1/5 (20%) with cirrhosis achieved sustained virological response (SVR). One of the four patients with cirrhosis that experienced virological failure carried the A30K RAS, and 2/4 patients with cirrhosis also carried the Y93H substitution. This finding suggested that these two RASs could have a major impact in compromised patients.

The sof/velpatasvir (sof/vel) trial (ASTRAL-3) [20] studied patients infected with GT-3 that had relapses. They found that 5/11 (45.4%) patients had baseline RASs: four had Y93H substitutions, and one patient previously treated with pegylated-interferon/ribavirin had the A30K substitution at baseline and at the 12-week follow-up. Another study by Pianko [21] evaluated patients that had received previous treatments for HCV infections. Among 52 patients with GT-3 cirrhosis that were treated with sof/vel, 8 (15.4%) had RASs at baseline, and of those, one (12.5%) showed no response to DAAs. Among 44 patients without RASs, 2 (4.5%) experienced treatment failure. However, no data on specific RASs were provided in that study. In phase III trials that evaluated patients with decompensated cirrhosis [22], of 39 patients infected with GT-3, 13 had a virologic failure response to sof/vel treatment, and of these, 3 (23%) patients displayed RAS Y93H before treatment, and 13/13 (100%) patients displayed this amino acid change after the treatment evaluation. In that trial, not all NS5A RAS positions were defined; consequently, it was not clear whether the RAS A30K substitution was absent or whether substitutions at position 30 were not analyzed.

A recent trial [23] (POLARIS-1) examined sof/vel/voxilaprevir treatment for patients with no response after previous treatments with DAA regimens, including NS5A-inhibitors. Of four patients infected with GT-3A that experienced a virologic relapse, three had a Y93H substitution at baseline or at relapse, and one patient had A30K at baseline and at virologic relapse. In the POLARIS-4 study, eight patients with GT-3A were previously treated with pegylated-interferon /ribavirin and had a virologic relapse. Of those patients, 7 had a Y93H substitution at baseline or at relapse, and one had a A30K substitution at baseline and experienced treatment failure.

In another study on glecaprevir and pibrentasvir, [24] one patient with a virologic breakthrough had a baseline A30K RAS that persisted to the time of failure. Another patient that relapsed at post-treatment week 2 had a baseline A30K RAS and displayed the emergence of Y93H at the time of failure. In another patient that experienced a virologic relapse at week 8, Y93H was present at baseline and at relapse.

Taken together, these data indicated that pre-existing NS5A RASs (A30K and Y93H) might affect virologic outcomes of patients with GT-3 infections and patients with more compromised conditions. It is not clear whether treatment should be shortened in the presence of NS5A RASs, due to the small sample size in the only study available [24]. Further evaluations are necessary in larger groups of patients infected with GT-3.

Our analysis showed that the A30L substitution was polymorphic in GT-3A from Asia, but it was absent in isolates from other geographic regions. To our knowledge, no “in vitro” data are available on the susceptibility of this mutant to NS5A inhibitors. Moreover, no data are available from clinical trials on the presence or susceptibility of GT-3A A30L variants to NS5A inhibitors. However, the majority of clinical trials on GT-3A have included non-Asian patients. Therefore, the clinical impact of this polymorphism at the site of resistance remains unknown.

Finally, we evaluated natural selective pressure of NS5A domain 1 region, by using the MEME algorithm, showing that in GT-3nonA isolates from Asia the position 30 was under selective pressure. Interestingly, this positive pressure was located in a position associated with resistance to NS5A inhibitors. We also observed that the codon 62 was under selective pressure in GT-3A, irrespective of geographic origin of isolates. The amino acid at position 62 has been located adjacent to Zn++ coordinate residues C57 and C59 according to the crystal structure of NS5A domain 1 from GT-1A [25]. In particular, Sun and coauthors showed that E62D substitution could be associated with resistance when in combination with Q30R [26]. However, a crystal structure of NS5a domain 1 is not available for GT3.

## 5. Conclusions

In conclusion, we demonstrated that the NS5A domain may be used in determining GT-3 subtypes and RASs simultaneously. We provided important information on the geographic distribution of naturally occurring GT-3 RASs that conferred resistance to NS5A inhibitors, according to different subtypes. We showed that the GT-3nonA subtypes occurred more frequently in Asian than in non-Asian isolates. We also showed that RAS A30K was the dominant virus in GT-3nonA isolates from Asia, and the A30L substitution was polymorphic in GT-3A isolates from the same geographic area. These peculiar characteristics of Asian GT-3A and GT-3nonA isolates are likely to support recommendations regarding baseline resistance testing and, possibly, treatment strategies in the near future.

## Figures and Tables

**Table 1:** Geographic distribution of GT-3 subtypes in Los Alamos HCV database.

	GT-3A	GT-3B °	GT-3G	GT-3H	GT-3K °°	GT-3I
Brazil (N = 597)	597	-	-	-	-	-
Australia (N = 18)	18	-	-	-	-	-
Europe (N = 86)	83	-	1	-	-	2
Pakistan (N = 25)	25	-	-	-	-	-
Thailand (N = 21)	18	3	-	-	-	-
China (N =16)	4	12	-	-	-	-
India (N = 16)	15	-	-	-	-	1
Other (N = 8)	3	-	1	1	1	2
TOTAL	763	15	2	1	1	5

° 1 GT-3B sequence was from Japan; °° 1 GT-3K sequence was from Indonesia.

**Table 2:** Mean fold change in resistance compared to wild-type replicon of RASs detected in GT-3a sequences retrieved by Los Alamos HCV database.

RASs	Replicative Capacity	DCV ° FC [8]	VEL °° FC [17]	PIB ** FC [16]
A30K	66	44	50	-
Y93H A30K + Y93H	34	2154	723	2.3

Mean fold change values for Daclatasvir were obtained by using bicistronic hybrid replicon JFH1/3ANS5A; mean fold change values for Velpatasvir were obtained by using GT-3A replicon S52 and mean fold change value for Pibrentasvir was obtained by using Con 1 chimeric replicon. ° DCV = daclatasvir, °° VEL = velpatasvir; ** PIB = pibrentasvir. FC = mean fold change in resistance compared to wild-type replicon. Empty cells indicate no data available from patients who experienced treatment failure. [] = References.

**Table 3:** Selective pressure analysis of NS5A domain 1 region according geographic origin and GT-3 subtypes.

Data Set	N	Site	alpha	beta-	p-	beta+	p+	LTR	*p*-Value
Overall	789	62	0.78	0.31	0.76	8.28	0.24	14.20	0.00
Asia	80	30	0.65	0.00	0.85	7.96	0.15	9.16	0.00
		62	1.37	0.25	0.73	10.35	0.27	6.83	0.01
GT-3A	62	62	0.98	0.00	0.72	12.20	0.28	6.90	0.01
GT-non3A	18	30	0.00	0.00	0.89	8.49	0.11	9.28	0.00
Other countries	709	62	0.63	0.51	0.91	13.26	0.09	5.10	0.04
GT-3A	701	62	0.63	0.57	0.87	11.03	0.13	6.40	0.02
GT-non3A	8 *	-	-	-	-	-	-	-	-

* None codon under selection, N = number of sequences, Site = site under selective pressure, alpha = synonymous substitution rate at site, beta- = non-synonymous rate at site for the negative/neutral evolution component, p- = proportion of the tree evolving neutrally or under negative selection, beta+ = nonsynonymous rate at site for the positive/neutral evolution component, p+ = proportion of the tree evolving neutrally or under positive selection, LTR = likelihood ratio test for episodic diversification p + > 0 and beta+ > alpha.

## Supplementary Materials

The following are available online at http://www.mdpi.com/1999-4915/11/2/148/s1, Table S1: Accession numbers of 709 non-Asian NS5a sequences included in the analysis, according GT-3 subtype and geographic origin, Table S2: Accession numbers of 80 Asian NS5a sequences included in the analysis, according GT-3 subtype and geographic origin.
