# Supplementary material for: Geographic Distribution of HCV-GT3 Subtypes and Naturally Occurring Resistance Associated Substitutions
Source: Viruses. 2019 Feb 11;11(2):148. doi: 10.3390/v11020148 (PMC6410151; doi:10.3390/v11020148)
Supplement: Supplementary file 1 [file viruses-11-00148-s001.pdf]

Table S1: Accession numbers of 709 non-Asian NS5a sequences included in the analysis, according GT-3 subtype and geographic origin. The isolates for which full genome sequence was available were in bold. The alphanumeric code for each 58 full genomes analyzed was shown in branches.

Accession number	GT-3 subtype	Country
FJ931541	3a	AUSTRALIA
FJ931542	3a	AUSTRALIA
FJ931543	3a	AUSTRALIA
FJ931545	3a	AUSTRALIA
FJ931546	3a	AUSTRALIA
FJ931548	3a	AUSTRALIA
FJ931549	3a	AUSTRALIA
FJ931550	3a	AUSTRALIA
FJ931552	3a	AUSTRALIA
FJ931553	3a	AUSTRALIA
FJ931554	3a	AUSTRALIA
FJ931558	3a	AUSTRALIA
FJ931560	3a	AUSTRALIA
FJ931562	3a	AUSTRALIA
FJ931563	3a	AUSTRALIA
FJ931566	3a	AUSTRALIA
FJ931567	3a	AUSTRALIA
FJ931568	3a	AUSTRALIA
EU826174	3a	BRAZIL
EU826175	3a	BRAZIL
EU826176	3a	BRAZIL
EU826177	3a	BRAZIL
EU826178	3a	BRAZIL
EU826179	3a	BRAZIL
EU826180	3a	BRAZIL
EU826181	3a	BRAZIL
EU826182	3a	BRAZIL
EU826183	3a	BRAZIL
EU826184	3a	BRAZIL
EU826185	3a	BRAZIL
EU826186	3a	BRAZIL
EU826187	3a	BRAZIL
EU826188	3a	BRAZIL
EU826189	3a	BRAZIL
EU826190	3a	BRAZIL
EU826191	3a	BRAZIL
EU826192	3a	BRAZIL

EU826193	3a	BRAZIL
EU826194	3a	BRAZIL
EU826195	3a	BRAZIL
EU826196	3a	BRAZIL
EU826197	3a	BRAZIL
EU826198	3a	BRAZIL
EU826199	3a	BRAZIL
EU826200	3a	BRAZIL
EU826201	3a	BRAZIL
EU826202	3a	BRAZIL
EU826203	3a	BRAZIL
EU826204	3a	BRAZIL
EU826205	3a	BRAZIL
EU826206	3a	BRAZIL
EU826207	3a	BRAZIL
EU826208	3a	BRAZIL
EU826209	3a	BRAZIL
EU826210	3a	BRAZIL
EU826211	3a	BRAZIL
EU826212	3a	BRAZIL
EU826213	3a	BRAZIL
EU826214	3a	BRAZIL
EU826215	3a	BRAZIL
EU826216	3a	BRAZIL
EU826217	3a	BRAZIL
EU826218	3a	BRAZIL
EU826219	3a	BRAZIL
EU826220	3a	BRAZIL
EU826221	3a	BRAZIL
EU826222	3a	BRAZIL
EU826223	3a	BRAZIL
EU826224	3a	BRAZIL
EU826225	3a	BRAZIL
EU826226	3a	BRAZIL
EU826227	3a	BRAZIL
EU826228	3a	BRAZIL
EU826229	3a	BRAZIL
EU826230	3a	BRAZIL
EU826231	3a	BRAZIL
EU826232	3a	BRAZIL
EU826233	3a	BRAZIL
EU826234	3a	BRAZIL
EU826235	3a	BRAZIL
EU826236	3a	BRAZIL
EU826237	3a	BRAZIL
EU826238	3a	BRAZIL

EU826239	3a	BRAZIL
EU826240	3a	BRAZIL
EU826241	3a	BRAZIL
EU826242	3a	BRAZIL
EU826243	3a	BRAZIL
EU826244	3a	BRAZIL
EU826245	3a	BRAZIL
EU826246	3a	BRAZIL
EU826247	3a	BRAZIL
EU826248	3a	BRAZIL
EU826249	3a	BRAZIL
EU826250	3a	BRAZIL
EU826251	3a	BRAZIL
EU826252	3a	BRAZIL
EU826253	3a	BRAZIL
EU826254	3a	BRAZIL
EU826255	3a	BRAZIL
EU826256	3a	BRAZIL
EU826257	3a	BRAZIL
EU826258	3a	BRAZIL
EU826259	3a	BRAZIL
EU826260	3a	BRAZIL
EU826261	3a	BRAZIL
EU826262	3a	BRAZIL
EU826263	3a	BRAZIL
EU826264	3a	BRAZIL
EU826265	3a	BRAZIL
EU826266	3a	BRAZIL
EU826267	3a	BRAZIL
EU826268	3a	BRAZIL
EU826269	3a	BRAZIL
EU826270	3a	BRAZIL
EU826271	3a	BRAZIL
EU826272	3a	BRAZIL
EU826273	3a	BRAZIL
EU826274	3a	BRAZIL
EU826275	3a	BRAZIL
EU826276	3a	BRAZIL
EU826277	3a	BRAZIL
EU826278	3a	BRAZIL
EU826279	3a	BRAZIL
EU826280	3a	BRAZIL
EU826281	3a	BRAZIL
EU826282	3a	BRAZIL
EU826283	3a	BRAZIL
EU826284	3a	BRAZIL

EU826285	3a	BRAZIL
EU826286	3a	BRAZIL
EU826287	3a	BRAZIL
EU826288	3a	BRAZIL
EU826289	3a	BRAZIL
EU826290	3a	BRAZIL
EU826291	3a	BRAZIL
EU826292	3a	BRAZIL
EU826293	3a	BRAZIL
EU826294	3a	BRAZIL
EU826295	3a	BRAZIL
EU826296	3a	BRAZIL
EU826297	3a	BRAZIL
EU826298	3a	BRAZIL
EU826299	3a	BRAZIL
EU826300	3a	BRAZIL
EU826301	3a	BRAZIL
EU826302	3a	BRAZIL
EU826303	3a	BRAZIL
EU826304	3a	BRAZIL
EU826305	3a	BRAZIL
EU826306	3a	BRAZIL
EU826307	3a	BRAZIL
EU826308	3a	BRAZIL
EU826309	3a	BRAZIL
EU826310	3a	BRAZIL
EU826311	3a	BRAZIL
EU826312	3a	BRAZIL
EU826313	3a	BRAZIL
EU826314	3a	BRAZIL
EU826315	3a	BRAZIL
EU826316	3a	BRAZIL
EU826317	3a	BRAZIL
EU826318	3a	BRAZIL
EU826319	3a	BRAZIL
EU826320	3a	BRAZIL
EU826321	3a	BRAZIL
EU826322	3a	BRAZIL
EU826323	3a	BRAZIL
EU826324	3a	BRAZIL
EU826325	3a	BRAZIL
EU826326	3a	BRAZIL
EU826327	3a	BRAZIL
EU826328	3a	BRAZIL
EU826329	3a	BRAZIL
EU826330	3a	BRAZIL

EU826331	3a	BRAZIL
EU826332	3a	BRAZIL
EU826333	3a	BRAZIL
EU826334	3a	BRAZIL
EU826335	3a	BRAZIL
EU826336	3a	BRAZIL
EU826337	3a	BRAZIL
EU826338	3a	BRAZIL
EU826339	3a	BRAZIL
EU826340	3a	BRAZIL
EU826341	3a	BRAZIL
EU826342	3a	BRAZIL
EU826343	3a	BRAZIL
EU826344	3a	BRAZIL
EU826345	3a	BRAZIL
EU826346	3a	BRAZIL
EU826347	3a	BRAZIL
EU826348	3a	BRAZIL
EU826349	3a	BRAZIL
EU826350	3a	BRAZIL
EU826351	3a	BRAZIL
EU826352	3a	BRAZIL
JN689511	3a	BRAZIL
JN689512	3a	BRAZIL
JN689513	3a	BRAZIL
JN689514	3a	BRAZIL
JN689515	3a	BRAZIL
JN689516	3a	BRAZIL
JN689517	3a	BRAZIL
JN689518	3a	BRAZIL
JN689519	3a	BRAZIL
JN689521	3a	BRAZIL
JN689522	3a	BRAZIL
JN689523	3a	BRAZIL
JN689524	3a	BRAZIL
JN689525	3a	BRAZIL
JN689526	3a	BRAZIL
JN689527	3a	BRAZIL
JN689528	3a	BRAZIL
JN689529	3a	BRAZIL
JN689530	3a	BRAZIL
JN689531	3a	BRAZIL
JN689532	3a	BRAZIL
JN689533	3a	BRAZIL
JN689534	3a	BRAZIL
JN689535	3a	BRAZIL

JN689536	3a	BRAZIL
JN689537	3a	BRAZIL
JN689538	3a	BRAZIL
JN689539	3a	BRAZIL
JN689540	3a	BRAZIL
JN689541	3a	BRAZIL
JN689542	3a	BRAZIL
JN689543	3a	BRAZIL
JN689544	3a	BRAZIL
JN689545	3a	BRAZIL
JN689546	3a	BRAZIL
JN689547	3a	BRAZIL
JN689548	3a	BRAZIL
JN689549	3a	BRAZIL
JN689550	3a	BRAZIL
JN689551	3a	BRAZIL
JN689552	3a	BRAZIL
JN689553	3a	BRAZIL
JN689554	3a	BRAZIL
JN689555	3a	BRAZIL
JN689556	3a	BRAZIL
JN689557	3a	BRAZIL
JN689558	3a	BRAZIL
JN689559	3a	BRAZIL
JN689560	3a	BRAZIL
JN689561	3a	BRAZIL
JN689562	3a	BRAZIL
JN689563	3a	BRAZIL
JN689564	3a	BRAZIL
JN689565	3a	BRAZIL
JN689566	3a	BRAZIL
JN689567	3a	BRAZIL
JN689568	3a	BRAZIL
JN689569	3a	BRAZIL
JN689570	3a	BRAZIL
JN689571	3a	BRAZIL
JN689572	3a	BRAZIL
JN689573	3a	BRAZIL
JN689574	3a	BRAZIL
JN689575	3a	BRAZIL
JN689576	3a	BRAZIL
JN689577	3a	BRAZIL
JN689578	3a	BRAZIL
JN689579	3a	BRAZIL
JN689580	3a	BRAZIL
JN689581	3a	BRAZIL

JN689582	3a	BRAZIL
JN689583	3a	BRAZIL
JN689584	3a	BRAZIL
JN689585	3a	BRAZIL
JN689586	3a	BRAZIL
JN689587	3a	BRAZIL
JN689588	3a	BRAZIL
JN689589	3a	BRAZIL
JN689590	3a	BRAZIL
JN689591	3a	BRAZIL
JN689592	3a	BRAZIL
JN689593	3a	BRAZIL
JN689594	3a	BRAZIL
JN689595	3a	BRAZIL
JN689596	3a	BRAZIL
JN689597	3a	BRAZIL
JN689598	3a	BRAZIL
JN689599	3a	BRAZIL
JN689600	3a	BRAZIL
JN689601	3a	BRAZIL
JN689602	3a	BRAZIL
JN689603	3a	BRAZIL
JN689604	3a	BRAZIL
JN689605	3a	BRAZIL
JN689606	3a	BRAZIL
JN689607	3a	BRAZIL
JN689608	3a	BRAZIL
JN689609	3a	BRAZIL
JN689610	3a	BRAZIL
JN689611	3a	BRAZIL
JN689612	3a	BRAZIL
JN689613	3a	BRAZIL
JN689614	3a	BRAZIL
JN689615	3a	BRAZIL
JN689616	3a	BRAZIL
JN689617	3a	BRAZIL
JN689618	3a	BRAZIL
JN689619	3a	BRAZIL
JN689620	3a	BRAZIL
JN689621	3a	BRAZIL
JN689622	3a	BRAZIL
JN689623	3a	BRAZIL
JN689624	3a	BRAZIL
JN689625	3a	BRAZIL
JN689626	3a	BRAZIL
JN689627	3a	BRAZIL

JN689628	3a	BRAZIL
JN689629	3a	BRAZIL
JN689630	3a	BRAZIL
JN689631	3a	BRAZIL
JN689632	3a	BRAZIL
JN689633	3a	BRAZIL
JN689634	3a	BRAZIL
JN689635	3a	BRAZIL
JN689636	3a	BRAZIL
JN689637	3a	BRAZIL
JN689638	3a	BRAZIL
JN689639	3a	BRAZIL
JN689640	3a	BRAZIL
JN689641	3a	BRAZIL
JN689642	3a	BRAZIL
JN689643	3a	BRAZIL
JN689644	3a	BRAZIL
JN689645	3a	BRAZIL
JN689646	3a	BRAZIL
JN689647	3a	BRAZIL
JN689648	3a	BRAZIL
JN689649	3a	BRAZIL
JN689650	3a	BRAZIL
JN689651	3a	BRAZIL
JN689652	3a	BRAZIL
JN689653	3a	BRAZIL
JN689654	3a	BRAZIL
JN689655	3a	BRAZIL
JN689656	3a	BRAZIL
JN689657	3a	BRAZIL
JN689658	3a	BRAZIL
JN689659	3a	BRAZIL
JN689660	3a	BRAZIL
JN689661	3a	BRAZIL
JN689662	3a	BRAZIL
JN689663	3a	BRAZIL
JN689664	3a	BRAZIL
JN689665	3a	BRAZIL
JN689666	3a	BRAZIL
JN689667	3a	BRAZIL
JN689668	3a	BRAZIL
JN689669	3a	BRAZIL
JN689670	3a	BRAZIL
JN689671	3a	BRAZIL
JN689672	3a	BRAZIL
JN689673	3a	BRAZIL

JN689674	3a	BRAZIL
JN689675	3a	BRAZIL
JN689676	3a	BRAZIL
JN689677	3a	BRAZIL
JN689678	3a	BRAZIL
JN689679	3a	BRAZIL
JN689680	3a	BRAZIL
JN689681	3a	BRAZIL
JN689682	3a	BRAZIL
JN689683	3a	BRAZIL
JN689684	3a	BRAZIL
JN689685	3a	BRAZIL
JN689686	3a	BRAZIL
JN689688	3a	BRAZIL
JN689689	3a	BRAZIL
JN689690	3a	BRAZIL
JN689691	3a	BRAZIL
JN689692	3a	BRAZIL
JN689693	3a	BRAZIL
JN689694	3a	BRAZIL
JN689695	3a	BRAZIL
JN689696	3a	BRAZIL
JN689697	3a	BRAZIL
JN689698	3a	BRAZIL
JN689699	3a	BRAZIL
JN689700	3a	BRAZIL
JN689701	3a	BRAZIL
JN689702	3a	BRAZIL
JN689703	3a	BRAZIL
JN689704	3a	BRAZIL
JN689705	3a	BRAZIL
JN689706	3a	BRAZIL
JN689707	3a	BRAZIL
JN689708	3a	BRAZIL
JN689709	3a	BRAZIL
JN689710	3a	BRAZIL
JN689711	3a	BRAZIL
JN689712	3a	BRAZIL
JN689713	3a	BRAZIL
JN689714	3a	BRAZIL
JN689715	3a	BRAZIL
JN689716	3a	BRAZIL
JN689717	3a	BRAZIL
JN689718	3a	BRAZIL
JN689719	3a	BRAZIL
JN689720	3a	BRAZIL

JN689721	3a	BRAZIL
JN689722	3a	BRAZIL
JN689723	3a	BRAZIL
JN689724	3a	BRAZIL
JN689725	3a	BRAZIL
JN689726	3a	BRAZIL
JN689727	3a	BRAZIL
JN689728	3a	BRAZIL
JN689729	3a	BRAZIL
JN689730	3a	BRAZIL
JN689731	3a	BRAZIL
JN689732	3a	BRAZIL
JN689733	3a	BRAZIL
JN689734	3a	BRAZIL
JN689735	3a	BRAZIL
JN689736	3a	BRAZIL
JN689737	3a	BRAZIL
JN689738	3a	BRAZIL
JN689739	3a	BRAZIL
JN689740	3a	BRAZIL
JN689741	3a	BRAZIL
JN689742	3a	BRAZIL
JN689743	3a	BRAZIL
JN689744	3a	BRAZIL
JN689745	3a	BRAZIL
JN689746	3a	BRAZIL
JN689747	3a	BRAZIL
JN689748	3a	BRAZIL
JN689749	3a	BRAZIL
JN689750	3a	BRAZIL
JN689751	3a	BRAZIL
JN689752	3a	BRAZIL
JN689753	3a	BRAZIL
JN689754	3a	BRAZIL
JN689755	3a	BRAZIL
JN689756	3a	BRAZIL
JN689757	3a	BRAZIL
JN689758	3a	BRAZIL
JN689759	3a	BRAZIL
JN689760	3a	BRAZIL
JN689761	3a	BRAZIL
JN689762	3a	BRAZIL
JN689763	3a	BRAZIL
JN689764	3a	BRAZIL
JN689765	3a	BRAZIL
JN689766	3a	BRAZIL

JN689767	3a	BRAZIL
JN689768	3a	BRAZIL
JN689769	3a	BRAZIL
JN689770	3a	BRAZIL
JN689771	3a	BRAZIL
JN689772	3a	BRAZIL
JN689773	3a	BRAZIL
JN689774	3a	BRAZIL
JN689775	3a	BRAZIL
JN689776	3a	BRAZIL
JN689777	3a	BRAZIL
JN689778	3a	BRAZIL
JN689779	3a	BRAZIL
JN689780	3a	BRAZIL
JN689781	3a	BRAZIL
JN689782	3a	BRAZIL
JN689783	3a	BRAZIL
JN689784	3a	BRAZIL
JN689785	3a	BRAZIL
JN689786	3a	BRAZIL
JN689787	3a	BRAZIL
JN689788	3a	BRAZIL
JN689789	3a	BRAZIL
JN689790	3a	BRAZIL
JN689791	3a	BRAZIL
JN689792	3a	BRAZIL
JN689793	3a	BRAZIL
JN689794	3a	BRAZIL
JN689795	3a	BRAZIL
JN689796	3a	BRAZIL
JN689797	3a	BRAZIL
JN689798	3a	BRAZIL
JN689799	3a	BRAZIL
JN689800	3a	BRAZIL
JN689801	3a	BRAZIL
JN689802	3a	BRAZIL
JN689803	3a	BRAZIL
JN689804	3a	BRAZIL
JN689805	3a	BRAZIL
JN689806	3a	BRAZIL
JN689807	3a	BRAZIL
JN689808	3a	BRAZIL
JN689809	3a	BRAZIL
JN689810	3a	BRAZIL
JN689811	3a	BRAZIL
JN689812	3a	BRAZIL

JN689813	3a	BRAZIL
JN689814	3a	BRAZIL
JN689815	3a	BRAZIL
JN689816	3a	BRAZIL
JN689817	3a	BRAZIL
JN689818	3a	BRAZIL
JN689819	3a	BRAZIL
JN689820	3a	BRAZIL
JN689821	3a	BRAZIL
JN689822	3a	BRAZIL
JN689823	3a	BRAZIL
JN689824	3a	BRAZIL
JN689825	3a	BRAZIL
JN689826	3a	BRAZIL
JN689827	3a	BRAZIL
JN689828	3a	BRAZIL
JN689829	3a	BRAZIL
JN689830	3a	BRAZIL
JN689831	3a	BRAZIL
JN689832	3a	BRAZIL
JN689833	3a	BRAZIL
JN689834	3a	BRAZIL
JN689835	3a	BRAZIL
JN689836	3a	BRAZIL
JN689837	3a	BRAZIL
JN689838	3a	BRAZIL
JN689839	3a	BRAZIL
JN689840	3a	BRAZIL
JN689841	3a	BRAZIL
JN689842	3a	BRAZIL
JN689843	3a	BRAZIL
JN689844	3a	BRAZIL
JN689845	3a	BRAZIL
JN689846	3a	BRAZIL
JN689847	3a	BRAZIL
JN689848	3a	BRAZIL
JN689849	3a	BRAZIL
JN689850	3a	BRAZIL
JN689851	3a	BRAZIL
JN689852	3a	BRAZIL
JN689853	3a	BRAZIL
JN689854	3a	BRAZIL
JN689855	3a	BRAZIL
JN689856	3a	BRAZIL
JN689857	3a	BRAZIL
JN689858	3a	BRAZIL

JN689859	3a	BRAZIL
JN689860	3a	BRAZIL
JN689861	3a	BRAZIL
JN689862	3a	BRAZIL
JN689863	3a	BRAZIL
JN689864	3a	BRAZIL
JN689865	3a	BRAZIL
JN689866	3a	BRAZIL
JN689867	3a	BRAZIL
JN689868	3a	BRAZIL
JN689869	3a	BRAZIL
JN689870	3a	BRAZIL
JN689871	3a	BRAZIL
JN689872	3a	BRAZIL
JN689873	3a	BRAZIL
JN689874	3a	BRAZIL
JN689875	3a	BRAZIL
JN689876	3a	BRAZIL
JN689877	3a	BRAZIL
JN689878	3a	BRAZIL
JN689879	3a	BRAZIL
JN689880	3a	BRAZIL
JN689881	3a	BRAZIL
JN689882	3a	BRAZIL
JN689883	3a	BRAZIL
JN689884	3a	BRAZIL
JN689885	3a	BRAZIL
JN689886	3a	BRAZIL
JN689887	3a	BRAZIL
JN689888	3a	BRAZIL
JN689889	3a	BRAZIL
JN689890	3a	BRAZIL
JN689891	3a	BRAZIL
JN689892	3a	BRAZIL
JN689893	3a	BRAZIL
JN689894	3a	BRAZIL
JN689895	3a	BRAZIL
JN689896	3a	BRAZIL
JN689897	3a	BRAZIL
JN689898	3a	BRAZIL
JN689899	3a	BRAZIL
JN689900	3a	BRAZIL
JN689901	3a	BRAZIL
JN689902	3a	BRAZIL
JN689903	3a	BRAZIL
JN689904	3a	BRAZIL

JN689905	3a	BRAZIL
JN689906	3a	BRAZIL
JN689907	3a	BRAZIL
JN689908	3a	BRAZIL
JN689909	3a	BRAZIL
JN689910	3a	BRAZIL
JN689911	3a	BRAZIL
JN689912	3a	BRAZIL
JN689913	3a	BRAZIL
JN689914	3a	BRAZIL
JN689915	3a	BRAZIL
JN689916	3a	BRAZIL
JN689917	3a	BRAZIL
JN689918	3a	BRAZIL
JN689919	3a	BRAZIL
JN689920	3a	BRAZIL
JN689921	3a	BRAZIL
JN689922	3a	BRAZIL
JN689923	3a	BRAZIL
JN689924	3a	BRAZIL
JN689925	3a	BRAZIL
JN689926	3a	BRAZIL
JN689927	3a	BRAZIL
JN689928	3a	BRAZIL
JN689929	3a	BRAZIL
JN689930	3a	BRAZIL
**JF735123**	**3g**	**CANADA (CA1)**
**JF735121**	**3h**	**CANADA (CA2)**
JF735125	3i	CANADA
**JX227969**	**3i**	**CANADA (CA3)**
**JF735122**	**3k**	**CANADA (CA4)**
AF320787	3a	EUROPE
AF320788	3a	EUROPE
AF320789	3a	EUROPE
AF320790	3a	EUROPE
AF320791	3a	EUROPE
AF320792	3a	EUROPE
AF320793	3a	EUROPE
AF320794	3a	EUROPE
AF320795	3a	EUROPE
AF320796	3a	EUROPE
AF320797	3a	EUROPE
AF320798	3a	EUROPE
AF320799	3a	EUROPE
AF320800	3a	EUROPE
AF320801	3a	EUROPE

AF320802	3a	EUROPE
AF320803	3a	EUROPE
AF320804	3a	EUROPE
AF320805	3a	EUROPE
AF320806	3a	EUROPE
AF320807	3a	EUROPE
AF320808	3a	EUROPE
AF320809	3a	EUROPE
AF320810	3a	EUROPE
AF320811	3a	EUROPE
AF320812	3a	EUROPE
AF320813	3a	EUROPE
**X76918**	**3a**	**EUROPE (EU29)**
**GU814263**	**3a**	**EUROPE (EU30)**
**DQ437509**	**3a**	**EUROPE (EU31)**
FJ931569	3a	EUROPE
FJ931571	3a	EUROPE
FJ931572	3a	EUROPE
FJ931574	3a	EUROPE
FJ931575	3a	EUROPE
FJ931576	3a	EUROPE
FJ931577	3a	EUROPE
FJ931578	3a	EUROPE
FJ931579	3a	EUROPE
FJ931585	3a	EUROPE
FJ931538	3a	EUROPE
FJ931539	3a	EUROPE
FJ931540	3a	EUROPE
FJ931587	3a	EUROPE
FJ931588	3a	EUROPE
FJ931590	3a	EUROPE
FJ931591	3a	EUROPE
FJ931592	3a	EUROPE
FJ931595	3a	EUROPE
FJ931596	3a	EUROPE
FJ931597	3a	EUROPE
FJ931598	3a	EUROPE
FJ931599	3a	EUROPE
FJ931600	3a	EUROPE
FJ931601	3a	EUROPE
FJ931603	3a	EUROPE
FJ931604	3a	EUROPE
FJ931605	3a	EUROPE
FJ931606	3a	EUROPE
FJ931607	3a	EUROPE
FJ931608	3a	EUROPE

FJ931609	3a	EUROPE
**GQ356200**	**3a**	**EUROPE (EU32)**
**GQ356201**	**3a**	**EUROPE (EU33)**
**GQ356202**	**3a**	**EUROPE (EU34)**
**GQ356203**	**3a**	**EUROPE (EU35)**
**GQ356204**	**3a**	**EUROPE (EU36)**
**GQ356205**	**3a**	**EUROPE (EU37)**
**GQ356206**	**3a**	**EUROPE (EU38)**
**GQ356207**	**3a**	**EUROPE (EU39)**
**GQ356208**	**3a**	**EUROPE (EU40)**
**GQ356209**	**3a**	**EUROPE (EU41)**
**GQ356210**	**3a**	**EUROPE (EU42)**
**GQ356211**	**3a**	**EUROPE (EU43)**
**GQ356212**	**3a**	**EUROPE (EU44)**
**GQ356213**	**3a**	**EUROPE (EU45)**
**GQ356214**	**3a**	**EUROPE (EU46)**
**GQ356215**	**3a**	**EUROPE (EU47)**
**GQ356216**	**3a**	**EUROPE (EU48)**
**GQ356217**	**3a**	**EUROPE (EU49)**
**JF509175**	**3a**	**EUROPE (EU50)**
**JF509176**	**3a**	**EUROPE (EU51)**
**JF509177**	**3a**	**EUROPE (EU52)**
**JX227954**	**3g**	**EUROPE (EU53)**
**JX227955**	**3i**	**EUROPE (EU54)**
**JX227956**	**3i**	**EUROPE (EU55)**
**AY956467**	**3a**	**UNITED STATES (US56)**
**DQ430819**	**3a**	**UNITED STATES (US57)**
**DQ430820**	**3a**	**UNITED STATES (US58)**

Table S2: Accession numbers of 80 Asian NS5a sequences included in the analysis, according GT-3 subtype and geographic origin. The isolates for which full genome sequence was available were in bold. The alphanumeric code for each 58 full genomes analyzed was shown in branches.

Accession number	GT-3 subtype	Country
**HQ639941**	**3a**	**CHINA (CN5)**
**HQ639942**	**3a**	**CHINA (CN6)**
**HQ912953**	**3a**	**CHINA (CN7)**
**KC844041**	**3a**	**CHINA (CN8)**
**JQ065709**	**3b**	**CHINA (CN9)**
KC441467	3b	CHINA
KC441468	3b	CHINA
KC441469	3b	CHINA
KC441470	3b	CHINA
KC441471	3b	CHINA
KC441472	3b	CHINA
KC441473	3b	CHINA
KC441474	3b	CHINA
KC441475	3b	CHINA
KC441476	3b	CHINA
**KC844044**	**3b**	**CHINA (CN10)**
**GQ275355**	**3a**	**INDIA (IN11)**
**HQ738645**	**3a**	**INDIA (IN12)**
**JN714194**	**3a**	**INDIA (IN13)**
**JQ717254**	**3a**	**INDIA (IN14)**
**JQ717255**	**3a**	**INDIA (IN15)**
**JQ717256**	**3a**	**INDIA (IN16)**
**JQ717257**	**3a**	**INDIA (IN17)**
**JQ717258**	**3a**	**INDIA (IN18)**
**JQ717259**	**3a**	**INDIA (IN19)**
**JQ717260**	**3a**	**INDIA (IN20)**
**KF035123**	**3a**	**INDIA (IN21)**
**KF035124**	**3a**	**INDIA (IN22)**
**KF035125**	**3a**	**INDIA (IN23)**
**KF035126**	**3a**	**INDIA (IN24)**
**KF035127**	**3a**	**INDIA (IN25)**
**FJ407092**	**3i**	**INDIA (IN26)**
**D63821**	**3k**	**INDONESIA (ID27)**
**D49374**	**3b**	**JAPAN (JP28)**
GQ300882	3a	PAKISTAN

GU294484	3a	PAKISTAN
KC415734	3a	PAKISTAN
KC415735	3a	PAKISTAN
KC415736	3a	PAKISTAN
KC415737	3a	PAKISTAN
KC415738	3a	PAKISTAN
KC415739	3a	PAKISTAN
KC415741	3a	PAKISTAN
KC415742	3a	PAKISTAN
KC415743	3a	PAKISTAN
KC415744	3a	PAKISTAN
KC415745	3a	PAKISTAN
KC415746	3a	PAKISTAN
KC415747	3a	PAKISTAN
KC415748	3a	PAKISTAN
KC415749	3a	PAKISTAN
KC415750	3a	PAKISTAN
KC415751	3a	PAKISTAN
KC415752	3a	PAKISTAN
KC415753	3a	PAKISTAN
KC415754	3a	PAKISTAN
KC415755	3a	PAKISTAN
KC415756	3a	PAKISTAN
KC415757	3a	PAKISTAN
HM042064	3a	THAILAND
HM042065	3a	THAILAND
HM042066	3a	THAILAND
HM042067	3a	THAILAND
HM042068	3a	THAILAND
HM042069	3a	THAILAND
HM042070	3a	THAILAND
HM042071	3a	THAILAND
HM042072	3a	THAILAND
HM042073	3a	THAILAND
HM042074	3a	THAILAND
HM042075	3a	THAILAND
HM042076	3a	THAILAND
HM042077	3a	THAILAND
HM042078	3a	THAILAND
HM042079	3a	THAILAND
HM042080	3a	THAILAND
HM042081	3a	THAILAND
HM042082	3b	THAILAND
HM042083	3b	THAILAND
HM042084	3b	THAILAND
